# Experimental animal models for diabetes and its related complications—a review

**DOI:** 10.1186/s42826-021-00101-4

**Published:** 2021-08-24

**Authors:** Chidhambara Priya Dharshini Kottaisamy, Divya S. Raj, V. Prasanth Kumar, Umamaheswari Sankaran

**Affiliations:** Department of Biotechnology, Manonmaniam Sundaranar University, Tirunelveli, 627 012 Tamil Nadu India

**Keywords:** Diabetes, Complications, Animal models, Alloxan, Streptozotocin

## Abstract

Diabetes mellitus, a very common and multifaceted metabolic disorder is considered as one of the fastest growing public health problems in the world. It is characterized by hyperglycemia, a condition with high glucose level in the blood plasma resulting from defects in insulin secretion or its action and in some cases both the impairment in secretion and also action of insulin coexist. Historically, animal models have played a critical role in exploring and describing malady pathophysiology and recognizable proof of targets and surveying new remedial specialists and in vivo medicines. In the present study, we reviewed the experimental models employed for diabetes and for its related complications. This paper reviews briefly the broad chemical induction of alloxan and streptozotocin and its mechanisms associated with type 1 and type 2 diabetes. Also we highlighted the different models in other species and other animals.

## Background

Diabetes is a chronic noncommunicable diseases (CNCDs) [1] characterized by chronic hyperglycemia resulting from defects in secretion and action of the pancreatic hormone insulin [2]. It is a group of metabolic diseases and considered as a leading global health problem [3]. In general, diabetes mellitus is categorized into type 1, type 2 and gestational diabetes based on the etiology and clinical features [4]. Type 1 diabetes was recently named adolescent diabetes or insulin-subordinate mellitus diabetes [5], a condition caused by the destruction of pancreatic β-cells by T cells resulting in a complete insulin deficiency [6]. Type 1 disease represents 5–10% of patients with diabetes [7]. Type 2 diabetes also known as adult onset diabetes or non insulin dependent diabetes is characterized by insulin resistance and relative insulin deficiency [8] and is the most prevalent form of diabetes accounting for at least 90% of all cases of diabetes [9]. Gestational diabetes occurs during pregnancy in women [10]. The fact behind this type of diabetes is that women with gestational diabetes are at an increased risk of developing type 2 diabetes later in their life [11]. The major cause for type 1 diabetes is believed to be a combination of genetic predisposition and additional environmental factors [12]. Type 2 diabetes is mainly caused due to insulin resistance [13]. Obesity is one of the leading causes for diabetes [14]. About 90% of the people with type 2 diabetes are obese [15]. Diabetes can also be caused due to damages to the pancreas [16] and also due to some rare genetic forms [17]. Diagnosis at an early stage and controlling blood sugar, blood pressure and cholesterol can prevent or delay the complications associated with diabetes [18]. The prevalence of diabetes in the Asian countries is higher particularly in India and China [19]. These two countries were ranked in the top two with 109.6 million and 69.2 million diabetics respectively [20]. In developed countries, about 10% of their budget for health care is utilized for the management of diabetes [21]. It has been predicted that the prevalence of diabetes mellitus will reach up to 642 million by the year 2040 globally [22]. Since diabetes imposes serious threat to individual’s health and causes societal economic burden [23], research on its management and treatment is now one of the most sought area of interest. In general, animal model study is essential for the development of new and effective means of treating diseases like diabetes. A number of animals are exploited for the experimental studies related to diabetes since animals are biologically similar with humans. Some of the animals employed in diabetes research and the methods of induction of diabetes in the animals are discussed below. The review also highlights the experimental animals employed for studying the diabetes related complications such as diabetic peripheral neuropathy, diabetic nephropathy and diabetic cardiomyopathy.

## Main text

### Animal models

In general, experimental diabetes mellitus is instigated in animals [24], because animal models plays an effective role in understanding the pathogenesis of the disease [25]. Even though a number of in vitro and in silico studies are available and are improved in the last decades, animal models still remains the effective one in understanding the complex etiology and multi-systemic interactions present in diabetes [26]. Many of the diabetes trials are performed in rodents while some studies are also done in larger animals. The experimental animal used in the study of diabetes mellitus can be categorized into three types such as genetically diabetic animals, miscellaneous models and other models based on the methods to induce experimental diabetes mellitus [27]. Diabetes can be developed in the experimental animals either by spontaneous methods or by using chemical agents [28]. Animal models may be developed by two principal mechanisms: disease induction (e.g., using specific drugs) or genetic manipulation. Both are of significant as they enable the analysis of particular mechanisms related to the disease and are important for understanding the pathogenesis and progression of the disease and extrapolating to humans. Since T1DM and T2DM are metabolic disorders that reflect complex integration of body systems, careful consideration is needed in choosing the correct animal model to be used in different in vivo experiments [29]. To achieve this goal, a careful analysis of the specific aspects of the disease and the specific knowledge that is targeted in each study must be performed when choosing a diabetes mellitus animal model [30]. The experimental animal models are classified based on the type of diabetes actually it mimics and also the mode of induction such as spontaneous or induced [26, 31]. Since T1DM is characterized by the deficiency of insulin production, the deficiency is achieved in experimental animals through chemical destruction of pancreatic β-cells or through breeding of rodents that spontaneously develop autoimmune diabetes. Onthe other hand, T2DM animal models are more numerous and may embrace obese and non-obese models with variable insulin resistance and β-cell failure degrees. In addition, there are transgenic and take out mouse models accessible, however their utilization in the examination field is as yet questionable [30].

### Mice and rats

Mice as an experimental animal have made enormous contributions to our understanding of human biology [32]. Mouse models are extensively exploited for studying the human disease because of the genetic homology between the two species [33]. The mouse models are extensively used to understand the basic knowledge of the human disease and the acquired knowledge progresses to preclinical investigations with the same mouse models [34]. With respect to diabetes, the mouse models are an invaluable one in obesity and type 2 diabetes experimental studies to identify the role of inflammation, insulin resistance, other potential treatments and the knowledge acquired from such studies are faithfully been carried out in humans diagnosed with such disease [35]. The rat as an experimental animal model of human disease offers various favourable circumstances and advantages over the mouse and different species [36]. The physiology in the rodent is simpler to follow and after some time an amount of information has developed which will take a very long time to recreate in the mouse [37]. Rat is extensively used as a suitable animal model for understanding the metabolic profile and pathology involved in different stages of type 2 diabetes [38]. A number of experimental mice and rat models are employed in the study of diabetes (Table 1) and are discussed below.

**Table 1:** Experimental rat and mice model for type 1 and type 2 diabetes

Method of induction	Model animal	Description	References
Chemical induction	Alloxan induced model	Selective inhibition of glucose stimulated insulin secretion	[41]
	Streptozotocin induced model	Damages the pancreatic β cell thereby causing hypoinsulinemia and hyperglycemia	[164]
Spontaneous auto immune	NOD mice	Polygenic model of Type 1 Diabetes characterized by hyperglycemia and leukocytic infiltration of the pancreatic islet of Langerhans	[11]
	BB rats	Spontaneously develop hyperglycemia and ketoacidosis that characterize the clinical onset of Type I Diabetes	[11]
	KDP rats	Spontaneous animal model with nonsense mutation in the Cblb and is a model of autoimmune type 1 diabetes	[68]
	LETL	Spontaneously developed autoimmune diabetes model without lymphopenia	[67]
	LEW-iddm	Spontaneously develops insulin dependent autoimmune diabetes through pancreatic β cell apoptosis	[11]
Genetically induced	AKITA mice	Genetically induced monogenic model that develops insulin dependent diabetes	[11]
	Zucker Diabetic Fatty rats	Developed with missense mutation in the leptin receptor gene. It develops obesity without diabetes and is used in the study of type 2 diabetes	[165]
	db/db	Diabetic model of type 2 diabetes having a mutation in the gene encoding leptin receptor	[165]
	GK rats	Polygenic model that develops adult onset type 2 diabetes earlier in their life	[166]
	Zucker fatty rats	Genetic obese model characterized by hyperlipidaemia and hypoinsulinemia	[167]
Genetically engineered	KK mouse	Polygenic diabetic model that exhibit type 2 diabetes associated with hyperglycemia, glucose intolerance and microalbuminaria	[168]
	Obese hyperglycemic mice	Used as Obesity model since these are overweight and hyperphagic from its young age and lack functional leptin	[167]
Surgical	Pancreatectomy model	Resemble type 2 diabetes since pancreatic beta cell mass gets reduced when certain percentage of pancreas is removed	[169]
Virus induced	Coxsackie B virus induced model	Develops insulin dependent diabetes mellitus as a result of re stimulation of resulting auto reactive T cells	[170]
	EMC virus induced model	Develops Diabetes mellitus by selective destruction of β cells	[171]

### Chemically induced rat and mice diabetic models

Some chemicals are used to induce diabetes in the experimental animals. Such chemicals are called as diabetogenic agents. Streptozotocin and alloxan are the commonly used chemical agents that induce diabetes when administered parenterally [39]. Depending on the animal species and route of administration, the dosage of the two drugs may vary [40].

### Alloxan induced models

Alloxan (5,5-dihydroxyl pyrimi-dine-2,4,6-trione) is an organic compound and is a cytotoxic glucose analogue [41] which is used to induce diabetes mellitus chemically by two proposed possible mechanisms [42]. One reports that alloxan specifically inhibits glucose-incited insulin emission by broad glucokinase restraint, the Beta-cell pancreatic glucose sensor [43] and it also induces reactive oxygen species (ROS) formation, creating a redox cycle generating superoxide radicals [44]. Alloxan is decreased to dialuric corrosive and then re-oxidized back to alloxane, making superoxide radicals that experience dismutation (by superoxide dismutase) to form hydrogen peroxide; side responses can likewise make hydroxyl radicals. These highly reactive oxygen species may cause β-cell DNA fragmentation leading to apoptosis [11, 43, 45]. While alloxan is additionally taken up by the liver, alloxan-prompted hepatotoxicity is insignificant or invalid on the grounds that the liver has more productive ROS safeguard instruments than β-cells [45]and they also have several mechanisms for xenobiotic biotransformation and elimination. Alloxan additionally advances basic oxidation—SH classes, particularly glutathione (GSH) compounds, proteins and likewise dysregulates intracellular calcium homeostasis bringing about centralizations of supraphysiological calcium and consequently cell harm [11, 45]. Dosages of alloxan extend from 50 to 200 mg/kg (in mice) and from 40 to 200 mg/kg (in rodents), contingent upon the strain and course of organization picked (e.g., intraperitoneal and subcutaneous organization of alloxane requires portions up to multiple times the intravenous organization [11].

### Streptozotocin induced models

Streptozotocin (STZ), chemically known as N- (methylnitrosocarbamoyl)-α-d-glucosamine is a naturally occurring compound produced by *Streptomycetes achromogenes* with antibacterial properties [46] that are selectively taken up by pancreatic β-cells causing its destruction [47]. It is also a cytotoxic glucose analogue [43] like alloxan. Rakieten reported the use of STZ as a diabetogenic [48]. The STZ is the most commonly used chemical for the induction of diabetes mellitus in the experimental animals [49]. It is a nitrosourea compound [50] with a toxic glucose and a N-acetyl glucosamine analogue that gets accumulated in the pancreatic β cells through the GLUT-2 (a transmembrane carrier protein) transporter uptake [51]. STZ induce diabetes rats, mice and other animals like rabbit and guinea pigs through two ways depending on the dose [52]. At high dose, STZ targets pancreatic β cells by its alkalyting property which is a normal function of the cytotoxic nitrosourea compounds [53]. In general, the nitrosourea compounds are lipophilic in nature and hence are easily uptaken by the cells, but in contrast the STZ being a nitrosourea compound is hydrophilic due to hexose substitution and are not easily uptaken by the cells thereby STZ is carried by a carrier protein of Glucose called GLUT-2 to the β cells because the chemical structure of the STZ resembles glucose moiety [54]. The β cell of the pancreas usually have selective properties of the STZ and hence the chemical compound keep α cell of the pancreas and the extra pancreatic cells in an intact condition and do not affect it [43]. It is the same in case with humans where STZ do not affect any of the pancreatic cells including β cell [46]. At low doses (usually given as multiple exposure), STZ induce immune and inflammatory response which is due to the release of the enzyme glutamic acid decarboxylase [55]. This enzyme is a major auto antigen in autoimmune diabetes [56]. When released from the islet β cell, the enzyme comes in contact with the immune effector cells [57]. This condition aids in the destruction of β cell and leads to the development of hyperglycemic state that is associated with inflammatory infiltrates in particular with lymphocytes of the pancreas [55]. In the high-portion STZ technique, a solitary portion of STZ is directed to mice by means of intravenous or intraperitoneal routes (100–200 mg/kg) or rats (35–65 mg/kg) producing massive pancreatic β-cell destruction with little or no insulin production [58]. Various low-portions STZ technique suggests that little dosages (20 to 40 mg/kg/day) ought to be directed over some undefined time frame to advance insulitis [11, 59].

### Spontaneous auto-immune rodents and mouse

NOD-mouse, diabetes prone BB rats, KDP rat, LETL rat and LEW-iddm rat are the widely used animal models of spontaneous diabetes for studying the autoimmune diabetes [28]. Nonobese diabetic (NOD) mouse is one of the most regularly utilized models for investigations of type 1 diabetes (T1D). This is due to the fact that NOD mouse resembles a number of genetic and immunological traits with the human form of the metabolic disorder [60]. Dissimilar to different models utilized in autoimmunity examines, this model can build up a comparative unconstrained sickness to humans. Utilization of this model has prompted a few advances in understanding the illness including recognizing a few auto antigens and biomarkers that are comparative in people and have empowered the development of therapeutic targets [61]. This NOD mouse originated in the interbreeding of Cataract Shionogi (CTS strain) expressed polyuria, glycosuria and lymphocytic infiltration in the islets of langerhans region of the pancreas [62]. The greatest hereditary factor adding to T1D defenselessness in both NOD mice and humans is the major histocompatibility complex (MHC). Like humans, many genes in the NOD mice are also susceptible to develop type 1 diabetes. The MHC alleles play an important role in the development of the disease. In humans and NOD mouse, the combined action of many MHC alleles with the non-MHC genes results in their diabetogenic action [28, 63]. The BB rats are the most valuable experimental animals for studying the genetic basis of type 1 diabetes [64] and also in intervention studies [65, 66]. This sort of rodent had been derived from the outbred Wistar rodents in which spontaneous hyperglycemia and ketoacidosis occurred in the 1970s period. From such affected rodents, two colonies were found that serves as the base for the establishment of all other BB rat colonies including one ingrained biobreeding diabetes-prone/worceste (BBDP/Wor) and one outbred biobreeding diabetes-prone (BBDP) rodent [67, 68]. Biobreeding (BB) rodents create diabetes after adolescence with a comparable rate among males and females with roughly 90% of rodents creating diabetes somewhere in the range of eight and seventeen years old. The diabetic phenotype is very serious and is portrayed by the improvement of hyperglycemia, hypoinsulinemia weight reduction and ketonuria [11]. Despite the fact that these animals have insulitis with T-cells, B-cells, macrophages and natural killer (NK) cells, they are lymphopenic with extreme decrease in CD4^+^T cells and close nonattendance of CD8^+^T cells. Lymphopenia is certainly not a quality of type 1 diabetes (T1D) either in people or Nonobese diabetic (NOD) mice [68]. The Komeda diabetes-prone (KDP) rat is one of the best spontaneous animal model of autoimmune type 1 diabetes disorder studies [69]. It is also an important experimental model in the study of autoimmune disorders in particular autoimmune thyroid disease [70]. Autoimmune destruction of pancreatic β cells and rapid onset of diabetes irrespective of age and sex difference and no significant T-lymphopenia are the phenotypic characterization of the KDP rats. The characteristic features are closely similar with human type 1 diabetes [71]. The KDP rats are associated with lymphocyte infiltration and most of the animals exhibit moderate to severe level of lymphocyte infiltration into the pancreatic islets (insulitis). About 80% of the animals develop diabetes within 220 days of their age [71]. The genetic analysis of type 1 diabetes in the KDP rats showed the genetic predisposition of diabetes which was explained by the two susceptible loci such as major histocompatibility complex (MHC) on chromosome 20 and IDDM/ KDP 1on chromosome 11 [72]. Later, Cblb was identified as a major susceptibility gene for type 1 diabetes of rat [70, 73]. The Long Evans Tokushima Lean (LETL) rats are one of the widely used spontaneous animal models of insulin dependent diabetes mellitus (IDDM) since it closely resemble the pathology of human IDDM [74]. These LETL rats were discovered in 1982 and originated from some pairs of outbred Long-Evans rat purchased from Charles river in Canada [75]. The phenotypic characterization of the LETL rats includes 1. Sudden onset of polyuria, polyphagia, hyperglycemia and weight loss. 2. Lymphocyte infiltration into the pancreatic islets (insulitis) which is followed by the destruction of β cells of the pancreas and at the onset of diabetes, the lymphocyte gets disappeared. 3. Lymphocyte infiltration into the salivary gland and lacrimal gland. 4. Severity of the disease irrespective of the age and gender difference. 5. Hyperplastic foci of pancreatic islets. 6. No significant lymphocytopenia. 7. Renal complications that include nodular lesions [27, 75, 76]. These characteristics are shown to be closely associated with human IDDM. The animals develop hyperglycemia after 18 weeks of their birth [27]. Genetic analysis of the animal showed that two recessive genes are necessitated in the pathogenesis of insulitis [74]. The LEW-iddm rodent model for diabetes (T1D) emerged spontaneously in a colony of Lewis congenic rodents portrayed by a characterized MHC Lewis.1AR1 (LEW.1AR1) haplotype. It is one of a widely used model for examining human type 1 diabetes T1D. These rodents obviously create diabetes between the ages of 60 and 90 days and are described by fast movement of insulitis prompting broad β cell destruction [77–79]. This animal develops type 1 diabetes through two modes with different rates of incidence. It develops autoimmune diabetes spontaneously at a rate of 2% (approximately) and through immunological perturbation at a rate that can reach a maximum of 100% [80]. This experimental rodent model develops diabetes with equal frequency in both male and females. This unique characterization differentiates it from other spontaneously induced models for studying type 1 diabetes [81]. The genetic analysis of the animal showed an autosomal recessive mode of inheritance for the diabetes inducing genes and it routes a path for the detailed characterization of the loci conferring diabetes [82].

### Genetically induced diabetes models

AKITA mice, GK rats, Zucker diabetic fatty rats, Obese spontaneously hypersensitive rat (SHR), ESS rats and diabetes mouse (db/db) falls under the category of genetically induced diabetes models. Of them, the most widely used genetically induced diabetic mouse models is AKITA mice. These mice have Ins2+/C96Y mutation which induce an irregular insulin folding and destruction of β cells [83, 84]. Initially, the Akita mice were originated on the C57BL/6 inbred strain in Akita, Japan. But these experimental animals were nowadays developed with various genetic backgrounds and are made commercially available in the market. This model involves chronic stress on protein processing involving the endoplasmic reticulum and unfolded protein response triggering apoptosis and diabetes. The unfolded protein response tries to compensate and reduce the protein load of the endoplasmic reticulum, increasing its folding capacity [85]. This leads to toxicity in pancreatic β cells, decreasing their insulin secretion. A significant raise in the glucose level and albuminuria is witnessed in 4 weeks of its age and albuminuria tends to increase at a higher rate during the 10th week. Hence are employed in the study of diabetic related complications. The Goto-Kakizaki (GK) rats are insulin-resistant and are non-obese. GK rodents are profoundly acquired strain of Wistar rodents which create type 2 diabetes immediately [86]. It is a genetic experimental model of type 2 diabetes and its related complications [87–89]. It develops peripheral insulin resistance after 56 days of their birth. It has decreased pancreatic β cells and its functions [90]. Disease progression of this rat is been associated with chronic inflammation and hence utilized in the study of pathophysiology and therapeutic studies of type 2 diabetes [91, 92]. The Zucker diabetic fatty (ZDF) rats are a type of experimental animal model that reflects type 2 diabetes of human form. These rats originated from a colony of outbred zucker rats in Walter Shaw’s laboratory in Indianapolis (USA). Genetic model of the zucker diabetic rat was established in 1991. Insulin resistance, hyperphagia and obesity occur in this animal as a result of spontaneous mutation of a simple autosomal recessive leptin receptor gene (fa) on chromosome 5 [93–96]. ZDF male rats are most widely employed for the study of type 2 diabetes and its progression from prediabetic to diabetic state [97]. The Obese spontaneously hypersensitive rat (SHR) is formed as a result of mutation and it exhibits genetic obesity, endogenous hyperlipemia and other metabolic abnormalities. This strain is formed by mating an unconstrained hypertensive female rodent of the Kyoto–Wistar strain with a normotensive Sprague–Dawley male. These models are widely employed for the study of relationship between endocrine and metabolic abnormalities to obesity. It is considered as an important animal model for the study of role of high blood pressure and hyperlipemia associated with the pathogenesis of arthrosclerosis [98, 99]. ESS rat (e Stilman Siagado) is an inbred rat line that is maintained in the school of medicine, Rosario University, Argentina. It is an experimental rat variety that develops mild diabetic syndrome that is not associated to obesity [100]. The animals experience unpredictable proportions of glucose resistance from 2 months old enough on. The condition is a gentle form of diabetes that doesn't reduce the life expectancy of animals. Half year old rodents exhibited pulverization of islet engineering and stroma fibrosis [101]. The diabetes mouse (db/db) is formed due to autosomal recessive mutation in the leptin receptor and is originated from the Jackson laboratory. The mutation occurs is a Gly to Thr mutation in the leptin receptor gene on chromosome 4. These animals are obviously obese which is evident from its 3–4 weeks of age, hyperglycemic which is usually observed during 4 to 8 weeks of its age and most importantly hyperphagic (excessive appetite) [102, 103]. An extreme diabetic condition happens in these mice and is described by the beginning stage of hypoinsulinemia and hyperglycemia. Leptin receptor changes bring about a broad phenotype indistinguishable from that of Ob mice. The leptin receptor (Ob-R) encodes five option joined forms, in particular Ob-Ra, Ob-Rb, Ob-Rc and Ob-Rd. The Ob-Rb transcript contains a supplement with an untimely stop codon in the C57BL/KsJ ob/ob mouse strain if the grafting is unusual [94].

### Genetically engineered diabetic mice

The genetically engineered diabetic mice include KK mouse and Obese hyperglycemic mice. The KK mouse is widely exploited for investigating the obesity-associated diabetes. The characteristic features of this mouse include moderate obesity, polyphagia and polyuria. The diabetic state of the KK mouse is found to be chemical diabetes since it showed glucose intolerance and insulin resistance but is not glycosuric and hyperglycemic [104]. The KK mouse has the capability to develop type 2 diabetes in response to high fat diet and aging [105]. Hereditarily fat mice (Obese hyperglycemic mice) were recognized by Bleisch et al. as having acquired diabetes. These mice are glycosuric, the level of non-fasting blood sugar is about 300 mg percent, but there is no ketonuria or coma [106]. Insulin resistance is one of its most interesting features. The langerhans islands are hypertrophic with increased insulin content. The diabetes condition of the human diabetic patient is obviously not quite the same as that of the hyperglycemic mice. Halaas et al. detailed that leptin substitution totally inverts the phenotype of obesity and diabetics [107].

### Surgical models

These models are widely employed for the study of regenerative capability of β cells or its progenitors. Partial pancreatectomy which involves the partial or total removal of pancreas through surgery is the model that are of greater importance for the study of diabetes. Removal of 95% of the pancreas causes diabetes in rat models within 3 months and a similar mechanism is found in dogs and pigs. But the fact is that 60% partial pancreatectomy does not cause increased blood glucose concentrations and a moderate increase in the β cell mass level is only witnessed [108]. About 90% pancreatectomy elicits moderate hyperglycemia which is later followed by the pancreatic regeneration [109]. The major drawback of this model is invasiveness (especially to healthy tissues rather than pancreas) which makes it technically insignificant [11].

### Virus induced models

Viruses cause diabetes mellitus through the degradation and infection of β cells in the pancreas. Numerous human infections used to actuate diabetes incorporate RNA picornoviruses, coxsackie B virus [110], kilham rat virus [111] and encephalomyocarditis virus (EMC) [112]. CVB4 (coxsackie virus B4) is the most predominantly found enterovirus in the diabetic individuals. The CVB strain isolated from pancreas of a diseased child diagnosed with diabetic ketoacidosis induce diabetes in murine cells when inoculated into it [113]. Coxsackie virus is associated with the development of insulin dependent diabetes mellitus*. *EMC-D virus can infect and destroy beta-cells of the pancreas in mice and cause hyperglycemia dependent on insulin. A clone of EMC virus is identified as NDK2. Intraperitoneal injection of NDK25 develops non insulin dependent diabetes mellitus [114].

### Other species with inherited diabetes symptom

#### Chinese hamster

Meier and Yerganian [114] described the occurrence of hereditary diabetes mellitus in the Chinese hamster (*Cricetulus griseus*). Diabetic hamsters have increased levels of blood sugar from a typical 110 mg to 600 mg each. Extreme polyuria, glycosuria, ketonuria, and proteinuria are the diabetic symptoms found in Chinese hamsters. Diabetic symptoms could be improved by administering insulin and oral antidiabetic drugs. Sections of the pancreas, liver and kidney show historically pathological changes. The number of pancreatic islets is decreasing and the remaining islet cells are abnormal [115].

#### TUCO-TUCO

Shrewd et al. detailed that the diabetic disorder in Tuco-Tucos (*Ctenomys talarum*) to be similar to sand rodents and prickly mice. Tuco-Tucos, however tend to be less hyperglycemic and are less prone to ketosis. Many animals mainly males become hyperphagic. The usual lesion in the pancreas is degranulation of β cell in few animals, but amyloid islet hyalinization has also been observed [116].

#### Sand rat

The sand rat (*Psammomys obesus*) lives in the north African and near east desert regions [117]. This rat model is used for studying the effects and consequences of diet and exercise in the development of type 2 diabetes [118]. The animals create diabetic side effects when fed up with laboratory chow rather than a full vegetable eating routine [119]. The diabetic disorder for the most part creates in sand rodents in 2–3 months. Truly hyperglycemic animals bite the dust rashly from the ketosis. These animals develop hyperlipidaemia and arthrosclerosis when fed up with cholesterol rich diet [120].

#### Spiny mouse

The spiny mouse (*Acomys cahirinus*) occurs in the eastern Mediterranean semi-desert areas. Under laboratory settings, diabetes occurs in around 15 per cent of animals. Diabetes is caused by endocrine-pancreatic hyperplasia. Some animals have obesity, moderate hyperglycemia and hypoinsulinemia and others have explicit glucosuric hyperglycemia that leads to fatal ketosis. Characteristically, all spiny mice have massive pancreatic islet hyperplasia and increased pancreatic insulin content [121].

#### African hamster

The spontaneous diabetes mellitus in the African hamster (*Mystromys albicaudatus*) is first described by Schmidt et al. The characteristic features of this species include hyperglycemia, polyuria, polyphagia, polydipsia, glucosuria, ketonuria and pancreatic lesions including β-cell vacuolization, glycogen infiltration, nuclear pycnosis, margination of organelles and β-cell death [27].

### Non rodent-diabetes models

#### Invertebrate animal model-*Bombyx mori*

A mammalian model can be replaced with an invertebrate animal model to overcome the problems associated with modern animal rights so that it can reduce the mortality rate of mammals [122]. Silkworms are established as an animal model for life sciences since it contains a number of genes that are homologous to humans. Besides this, the silkworm is moderately sized organisms that can be dissected easily. This feature enables the broad availability of silkworm as a model to perform oral administration and intravenous injection examination studies [123]. In humans, adenylate protein kinase signalling pathway is responsible for the regulation of blood glucose and the same signalling pathway also regulates hemolymph glucose levels in silkworm. Moreover, the insulin like peptide that is encoded by the genes of the silkworms shows 40% similarity with that of human insulin. These two features aid in the development of silkworm as a model for diabetes. This can be achieved through the expression of human insulin receptor (hIR) in transgenic silkworm [124].

#### Pigs as diabetic model

Pigs are employed as an excellent model for the study of diabetes and its related complications. It is because of the fact that the morphology of the pancreas of the pig and its overall metabolic status are similar with that of humans. The Yucatan pig is originally from Mexico's Yucatan Peninsula. The early origination and characterization of the animal occurred at Colorado State University. Reportedly, selective breeding is an effective method for raising pigs with reduced glucose tolerance [125]. Recently a yucatan mini pig model of diabetic dyslipidemia at the University of Missouri at Columbia has been described in which alloxan (175 mg/kg intravenously) was given to induce diabetes [126]. These pigs are found to have a normal number of islets of langerhans and β cells and also have normal insulin release in response to isoproterenol challenge.

#### Primate model-obese Rh monkey

Rhesus monkey as an experimental model for diabetes is initially described by Hansen et al. where the monkeys are classified into sequential phases of the metabolic disease based on some parameters like age, body weight, glucose tolerance, fasting insulin levels and secretory insulin levels. The heterogeneity of plasma insulin and blood glucose level is identified as sequential changes that show the development of impaired glucose tolerance and type 2 diabetes. Once the monkey become diabetic, an abundant level of islet amyloid formed sequentially [127]. Rhesus monkey fed up with a high fructose diet is found to produce insulin resistance, obesity and inflammations within a short span of time and finally develops type 2 diabetes [128]. The rhesus and cynomolgus monkeys are identified as useful animal models for investigating the effects of therapeutic arbitrations as a result of preclinical islet transplantation studies [129].

#### Zebra fish models

Nowadays, zebra fish is widely employed for diabetes research. It is an attractive model system to study the metabolic disorders and is also used to identify and develop treatment for such disorders. The zebra fish possess conserved cholesterol metabolism and energy homeostasis. These key features make them an ideal model for the study of lipid metabolism [130]. Moreover, the zebra fish have a well developed organ system like digestive system, skeletal muscle and adipose tissue similar to that of humans and hence are mostly utilized for the clinical studies [131]. The zebra fish when fed up with excessive nutrients is found to develop increased plasma triglyceride levels and are found to develop hepatic steatosis [132]. It is also used in the therapeutic studies of diet induced glucose intolerance and insulin resistance. The zebra fish possess high human genetic homology that aids in the exploitation of it for the study of metabolic syndromes. Because of the availability of its fully sequenced genome, easier genetic manipulation and higher fecundity rates, the zebra fish are established as a unique model for the study of metabolic disorders of humans [133].

#### Diabetic complications

Hyperglycemia, a condition characterized by high level of glucose in the blood is a characteristic feature of both type 1 and type 2 diabetes. Because of its subtle and chronic nature, the hyperglycemia plays a key role in the development of diabetic complications [134]. The complications associated with diabetes are categorized into two such as micro vascular which occurs when there is any damage to the blood vessels and macro vascular disease that usually occurs due to damage to arteries. It is found that the underlying mechanism behind the pathogenesis of such diabetic complication is oxidative stress that is formed by the overproduction of reactive oxygen species (ROS) [135]. It may also be due to any defects in the insulin signal transduction pathway that is a key pathway involved in maintaining glucose homeostasis. A bioactive sphingolipid called ceramide is found to have an inhibitory effect over such pathway and may lead to complications [136]. The complications related to diabetes include neuropathy, nephropathy, retinopathy and cardiovascular diseases [137]. A number of animal models are employed for investigating the diabetic complications (Table 2) and are discussed below.

**Table 2:** Experimental animal models for diabetic complications

Diabetic complications	Animal Model	Characterization	References
Diabetic neuropathy	STZ induced rat model	Reduced fibre size of the peroneal nerve and axon than that of the myelin sheath with impaired motor function	[139]
	C57BL/KS (db/db) mice	Decreased sensory nerve conduction velocity and density of intraepidermal nerve fibers (IENF)	[140]
	Ischemic reperfusion injury model	Decreased serum IL-10 level and nerve conduction velocity and nerve fibre density	[141]
	Chinese Hamster	Reduced conduction velocity	[142]
	Obese Rh Monkey	Reduced conduction velocity and prolonged duration of F-wave latencies	[143]
Diabetic nephropathy	NOD mice	Enlarged glomeruli and mesangial sclerosis	[146]
	C57BL/6	Albuminuria and reduced renal functions	[147]
	GK rat	Thickening of glomeruli leading to glomerular hypertrophy	[148]
	Zucker diabetic fatty rat	Glomerulosclerosis, tubulointerstitial fibrosis and renal hypertrophy	[149]
	Zebra fish	Over expression of CIN85/RukL causing edema	[150]
Diabetic retinopathy	Alloxan induced model	Microaneurysms with increased acellular capillaries	[153]
	Akita mice	Decreased number of amacrine and ganglion cells	[154]
	db/db mouse	Reduced number of Retinal ganglion cells with thickened retina	[153]
	Surgical model	Formation of proliferative and contractile cellular membranes in the retina	[155]
	Zebra fish	Degradation and thinning of retina	[156]
Diabetic cardiomyopathy	Alloxan induced model	Formation of advanced glycation end products leading to oxidative stress	[158]
	BB rats	Reduced calcium—stimulated ATPase activity and cardiac contractility	[159]
	OLETF rats	Alteration in left ventricular diastolic function	[161]
	STZ induced model	Fibrosis and apoptosis leading to myocardial damage	[162]
	GK rats	Hyperglycemia, hyperlipidaemia and cardiac cell death	[163]

#### Diabetic neuropathy

Diabetic neuropathy is characterized by peripheral nerve dysfunction especially in type 2 diabetic patients. About two third of diabetic patients are having clinical or subclinical diabetic neuropathy complication [138]. A downstream metabolic cascade that is usually formed as a consequence of chronic hyperglycemia leads to the peripheral nerve dysfunction in diabetic patients. The cascade may be formed through an increased flux of the polyol pathway that metabolize the unused glucose in the presence of enzymes and hence is an enzymatically driven pathway. Excessive release of cytokines, activation of protein C kinase and overstate oxidative stress that occurs as a result of hyperglycemia are also key features behind the development of peripheral nerve dysfunction [139].

#### Animal models for diabetic neuropathy

STZ induced rat model is used in the study of diabetic neuropathy. With the induction of STZ in rat, the fibre size of the peroneal nerve along with the axon size gets reduced twice than that of the myelin sheath. These changes lead to the understanding of motor conduction velocity found in diabetics and hence are utilized in investigating the development, progression and therapeutic options of diabetic neuropathy [140]. The C57BL/Ks (db/db) mice model is extensively used in the study of diabetic neuropathy. This model develops symptoms like decreased sensory nerve conduction, velocity and density of intraepidermal nerve fibers (IENF) also gets reduced when fed up with a high fat diet (24% fat, 24% protein and 41% carbohydrates) for a period of 12 weeks. These symptoms showcase the onset of diabetic neuropathy and hence these models are exploited for investigating the onset and progression of diabetic neuropathy [141]. Muthuraman et al. showed that animal model developed of ischemic-reperfusion injury inducednociceptive sensory neuropathy is effective in the study of pathophysiological mechanisms involved in the development of neuropathic pain because the model showed a decreased serum IL-10 level and nerve conduction velocity that is similar with humans [142]. The Chinese hamster are also well utilized in the study of diabetic neuropathy since it shows a reduction in the conduction velocity when diabetes is induced and such reduction is similar with the condition of diabetic neuropathy in humans [143]. The obese Rhm monkey model is exploited to understand the pathogenesis and therapeutic options of diabetic neuropathy since it showed reduced conduction velocity and prolonged duration of F-wave latencies evoked by electrical stimulation of the peripheral motor. Such changes are similar with humans and hence are considered as a primate model of human diabetic neuropathy [144].

#### Diabetic nephropathy

Diabetic nephropathy develops in 30 to 40% of diabetic patients and is due to hyperglycemia and presence of genetic predisposition. Hyperglycemia induces the protein C kinase activation and increase the production of glycosylation end products. Apart from this, hyperglycemia also stimulates the renal cells (both resident and non-resident) in producing cytokines, growth factors and humoral mediators and causes some structural and functional alterations finally leading to the shear stress. Through these effects, hyperglycemia leads to renal damage and is considered as a major complication of diabetes [145]. Multigenetic predisposition plays as an important role in the development of the complication and hence is called as complex genetic disease and a number of genes are involved in the development of the complication. Apart from genetic factors, epigenetic and environmental factors also play a lead role in the progression of diabetic nephropathy [146].

#### Animal models for diabetic nephropathy

A strain of nonobese, non diabetic mice developed together with diabetic strain of NOD mice is found to have spontaneous lipid deposition in the glomerular capillary lumina and also develops features of renal injury including enlarged glomeruli and mesangial sclerosis that are characteristic of human diabetic nephropathy [147]. Progressive albuminuria and vast decrease in renal function which are the characteristic of diabetic nephropathy are observed following induction of Akita mutation in the C57BL/6, DBA/2, and 129/SvEv strains and hence are broadly utilized in the study of diabetic nephropathy [148]. The GK rat model develops changes in the glomeruli (thickening) and tubular basement membrane and also develops glomerular hypertrophy. These features are observed in case of human diabetic nephropathy and hence are widely exploited for elucidating the pathogenesis and examining the novel therapies for diabetic nephropathy [149]. Zucker diabetic rats are also exploited in the study of diabetic nephropathy. As their age progress (week by week), this animal model develops renal hypertrophy and other pathological changes like glomerulosclerosis, tubular cell damage, tubulointerstitial fibrosis and inflammation that resemble the human diabetic nephropathy and therefore ZDF rats are useful rodent model of diabetic nephropathy [150]. Hyperglycemia induced in zebra fish through intraperitoneal injection of STZ develops changes like thickening of glomerular basement membrane and over expression of CIN85/RukL causing edema and disrupts the filtration barrier and so such models are employed for the study of diabetic nephropathy [151].

#### Diabetic retinopathy

In diabetic retinopathy also, the hyperglycemia plays an important role. Hyperglycemia is associated with retinal micro vascular damage and aids in the development and progression of diabetic retinopathy. The risk of developing diabetic retinopathy is closely dependent on factors such as blood glucose levels, blood pressure, lipid profiles and also on type and the span of diabetic disorder [152]. Like the above discussed complications, oxidative stress, increased polyol pathway, protein C kinase activation and accelerated formation of glycation endproducts plays a key role in the development of the complication. In addition to the above features, increased expression of vascular endothelial growth factor (VEGF), insulin like growth factor-1 (IEF-1) and activation of the renin–angiotensin–aldosterone system (RAAS) also developed as a consequence of hyperglycemia which in turn aids in the development of diabetic retinopathy [153].

#### Animal models for diabetic retinopathy

Alloxan is used to induce diabetic retinopathy in rats, mice, pigs and rabbits. It has been studied that alloxan induced mice develop loss of pericyte and retinal ganglion cells (RGCs) within 7 days of induction. The same model develops microaneurysms with increased acellular capillaries. With such broad range of efficiencies, the alloxan induced experimental mice and rats are employed in the investigational study of diabetic retinopathy [154]. Akita mice are employed as a model for early stage of diabetic retinopathy. A significant increase in the vascular permeability is witnessed at 12 weeks after hyperglycemia. At 3 months, the mice also exhibit thinning of the retina, cone loss, disturbance of synaptic connectivity and finally a decrease in the number of amacrine and ganglion cells [155]. Diabetes mouse (db/db) is exploited for the study of late stages of diabetic retinopathy since it presents reactive gliosis along with leakages of the vessels. The animal model usually shows a reduction in the number of retinal ganglion cells (RCGs) with increased thickness in the central retina of the eye [154]. Apart from that, pig eyes are employed in the research since it closely resembles the size, structure of the retina and vasculature of the human eyes. A model developed through proliferative vitreoretinopathy involving surgical methods and intravitreal injection of retinal pigment epithelial cells is used in the study of diabetic retinopathy [156]. The features like degradation and thinning of retina (aneurism like structure) are observed in high glucose incubation zebra fish models. Hyperglycemias induced in such models are found to have adverse effect on retina of the eye that are similar with humans. With such context, the zebra fish are also employed in the study of diabetic retinopathy [157].

#### Diabetic cardiomyopathy

Cardiovascular diseases in particular heart attack are the leading cause of complications in diabetes. The clinical outcome associated with heart failure is higher in patients with diabetes than in non diabetic patients. Diabetic cardiomyopathy is mainly associated with type 2 diabetes. This is due to the fact that incompetence to increase glucose metabolism, incapability in substrate utilization within the diabetic heart formed as a consequence of type 2 diabetes will develop uncoupling of mitochondria, glucotoxicity and lipotoxicity. This sequence of events initiates cardiac dysfunction that includes myocardial fibrosis and steatosis, remodelling of the extra cellular matrix, diastolic dysfunction and finally systolic dysfunction which progressed and finally leads to heart attack [158].

#### Animal models for diabetic cardiomyopathy

Alloxan induced diabetic model is employed in the investigation of therapeutic options for treating diabetic cardiomyopathy (DCM). Bhatti*et al* studied the effect of ethanolic extract of *Aegle marmelos* (L.) Correa (Rutaceae), a deciduous plant on early stage diabetic cardiomyopathy in alloxan induced diabetic rats. Conditions like hyperglycemia and excess of glucose along with structural alterations in collagen results in the formation of advanced glycation end (AGE) products which inturn cause oxidative stress in the tissue. Since the heart has low levels of free radical scavenging mechanisms, the excessive formation of reactive oxygen species results in the formation of cardiovascular complications in the rat [159]. BB rats are widely used in the study of diabetic cardiomyopathy since it develops lower calcium-stimulated ATPase activity, cardiac contractility and depressed left ventricular developed pressure that are seen during diabetics in humans [160]. Yucatan mini pig, a non rodent animal model is also employed in the study of diabetic cardiomyopathy. The Yucatan mini pig develops obesity and insulin resistance when overfed with western diet. The chemically induced diabetes in pigs are used to study the pathogenesis of cardiovasculopathies [161]. Apart from the above models, the Otsuka Long-Evans Tokushima Fatty (OLETF) rats are now established as a model of diabetic cardiomyopathy. The OLETF rats showed alteration in the left ventricular diastolic function in its prestage of type 2 diabetes with prolonged deceleration time and decreased amplitude of peak velocity of the early diastolic filling wave. These changes occur due to metabolic abnormalities without any difference in their blood pressure and heart rate. Hence the OLETF rats are proposed to be an experimental animal model for the study of mechanism of alteration of heart function [162]. STZ induced type 1 diabetes in spontaneously hypertensive rats (SHR rats) in over term develops myocardial damage characterized by fibrosis and apoptosis. Reduction of proinflammatory factors and reduced expression of antioxidant molecules are found to be responsible for the mechanism behind the damage [163]. With such mechanisms, the STZ induced diabetes models are also used in the study of cardiomyopathy. Untreated GK rats induced with diabetes showed hyperglycemia, hyperlipidaemia and finally cardiac cell death in its progressive stage and hence are also employed in the study of diabetic cardiomyopathy [164].

## Conclusions

A number of type 1 and type 2 diabetic animal models and models employed for studying the diabetic complications were described above. Each differs in their characteristic and the development of the disorder. The experimental models are widely employed in studying the pharmacology and mechanisms underlying the metabolic disorder. To explicate the pathogenesis of human diabetes and its complications such as retinopathy, nephropathy, cardiomyopathy and neuropathy, diabetic models are playing a key role as the prevalence and complication of this disease is increasing worldwide. Despite all the advantages these animals offer for investigating and developing novel and plausible drugs, they possess individual limitations that will also limit the design of new drugs and therapeutic interventions. Hence forth the selection of model is important that it should possess all the characteristics necessary to be of a perfect model that varies depending on the diabetic complications and animal of choice. A model of autoimmunity is generally preferred for studying the type 1 diabetes but for the type 2 diabetes animals that are obese, non-obese, hyperglycemic, insulin resistance and β-cell resistance are generally employed. Since the experimental animal models differ in their physiological pertinent and are employed to represent the diverse complications of human diabetic patients much care and study is necessary in selecting the appropriate models for each study.

## Data Availability

All data associated with the review were mentioned in the Manuscript.
